# Adverse pregnancy outcomes, ‘stillbirths and early neonatal deaths’ in Mutare district, Zimbabwe (2014): a descriptive study

**DOI:** 10.1186/s12884-019-2229-3

**Published:** 2019-03-06

**Authors:** Blessmore Vimbai Chaibva, Steve Olorunju, Simon Nyadundu, Andy Beke

**Affiliations:** 1 School of Health Systems and Public Health, Faculty of Health Sciences, University of Pretoria, HW Snyman Building (North), 31 Bophelo Road, Gezina, Pretoria, South Africa; 2 Biostatistics Unit, Medical Research Council of South Africa, Cape Town, South Africa; 3 Manicaland Provincial Medical Directorate, Ministry of Health and Child Care, 24C Avenue, Box 323, Mutare, Zimbabwe

**Keywords:** Perinatal deaths, Adverse pregnancy outcomes, Mutare district, Stillbirth, Zimbabwe

## Abstract

**Background:**

Perinatal deaths account for 7% of the global burden of disease, with developing countries contributing about 98% of deaths. The aim of this study was to describe the prevalence and factors contributing to adverse pregnancy outcomes, particularly perinatal death, among women at Sakubva hospital, Mutare district, Zimbabwe from January to June 2014.

**Methods:**

We conducted a retrospective review of 346 patient records, of women who delivered at Sakubva hospital and those referred from Mutare district facilities to Mutare Provincial Hospital, between January and June 2014. Descriptive statistics was used to explore the contributors to stillbirths and early neonatal deaths in Mutare.

**Results:**

Of the 346 women, 54 (15.61%) experienced an adverse pregnancy outcome (stillbirth or early neonatal death). Contributing factors to adverse pregnancy outcomes included birthweight, gestational age, delivery complications and delivery methods. These factors are preventable if quality focused antenatal care, intrapartum care is provided. Identification of pregnancy complications and facilitation of proper method of delivery is key to improve quality of care. Caesarean section provision to all women who need it improves outcomes.

**Conclusions:**

High prevalence of adverse pregnancy outcomes in Mutare district could be reduced through the provision of quality antenatal care throughout the continuum of care, pre-, intra and postpartum. Further studies to explore risk factors associated with high adverse outcomes are recommended.

## Background

Worldwide, nearly 3 million third trimester stillbirths occur annually and a similar number of children die within the first 28 days of life [1, 2]. These deaths account for approximately 7% of the global burden of disease, which is higher than that of HIV/AIDS [3]. Low and middle-income countries bear 98% of the burden with sub- Saharan Africa reporting the highest burden globally [4, 5]. In sub-Saharan Africa about 14% of all births could result in stillbirths [6]. Most deaths (45%) among under-fives occur within the first month of life (the neonatal period) [7].

Zimbabwe’s 2010/11 Demographic Health Survey (ZDHS) reported an infant mortality rate of 57 deaths per 1000 live births while the overall under-5 mortality rate for the period was 84 deaths per 1000 live births. The neonatal mortality ratio (NMR) has increased from 33 to 39 deaths per 1000 live births from 1990 to 2012 [8]. Zimbabwe Multiple Indicator Cluster Survey (MICS) 2014 also showed an upward trend in NMR from 20 deaths per 1000 live births in 2000 to 29 deaths per 1000 live births in 2014 [9]. The NMR in Zimbabwe is rising despite various interventions such as Results Based Financing which resulted in the removal of direct user fees for maternal services. Peer reviews of maternal and perinatal audits were introduced to improve the quality of services throughout the management of pregnancy. These efforts may be contributing towards the overall downward trend in under-five mortality [9]. Sustainable continuous improvement efforts to reduce newborn, child and maternal mortality should be put in place to end all such preventable deaths before 2030 [10].

Risk factors associated with adverse pregnancy outcomes can be categorized into socio-demographic factors, maternal factors, previous pregnancy outcomes, neonatal factors, socio-economic and health system related factors [11–16]. Data on the prevalence and determinants of adverse pregnancy outcomes in sub-Saharan Africa is sparse. The aim of this study was to describe the prevalence of adverse pregnancy outcomes (stillbirths and early neonatal deaths) in Mutare district for the period January to June 2014. The specific objectives were to categorize the factors into socio-demographic, neonatal, previous pregnancy outcomes and maternal factors.

## Methods

### Study design and setting

Manicaland is the country’s second-most populous province with a population of 1,752,698 (2012 Census) constituting approximately 13.4% of the country population. Most Zimbabweans, 67%, live in rural areas. According to DHIS2 data January to December 2013, the province recorded 1540 perinatal deaths, against 44,610 (42,875 Institutional and 1735 home). In the same year, Mutare district recorded 534 (stillbirths + ENND) against 9673 (institutional live births) which translated to 55.2 deaths per 1000 live births [17]. However, this could be an underestimate due to the poor vital registry system.

Mutare district population (35.7% of Manicaland) is served by a network of 48 primary, 1 secondary (Sakubva) and 1 tertiary (Mutare Provincial) health centres. For the period January to June 2013 (DHIS2 data): Manicaland province recorded 493 adverse pregnancy outcomes, against 20,869 deliveries while Mutare district recorded 4690 births (5497 expected births) and 197 adverse pregnancy outcomes (stillbirths and fresh neonatal deaths). Thus, Mutare district contributed close to 40% of the adverse pregnancy outcomes [17].

Data were sourced from birth registers of Sakubva and Mutare Provincial Hospitals in Manicaland Province, Zimbabwe. Women resident in Mutare district, aged 18 years and above who had a singleton birth during the period January to June 2014 were included in the study. Records with more than 20% missing data were excluded.

Primary health facilities (PHF) manage non-complicated Antenatal (ANC), deliveries and postnatal cases. All para 0, under 18 pregnant women and those who have had previous caesarean sections, have ANC monitoring at clinic but are referred to district hospitals for delivery. For emergency referral, facility calls the district hospital to request an ambulance and if ambulance is not readily available, local transport is hired for transfer of patient. Physicians at Sakubva manage cases and refer complicated cases to Mutare provincial hospital for management by gynaecological specialists. All referrals from Sakubva were followed up at Mutare Provincial Hospital and considered for the study.

About 20% of women in Zimbabwe deliver at home or in the community (ZDHS 2015 report). Home deliveries have been attributed to perceived low economic, social and cultural opportunity costs, religious beliefs, culture, influence of family members, fear of hospital procedures, accessibility, and perceived high costs. Community maternal providers allure home delivery as they are perceived to be cheaper, have flexible payment terms and promote family cohesion thereby provide sensitivity to cultural and religious preferences [18].

We conducted a descriptive study on data extracted from the “delivery register” from the period January 1st to June 30th, 2014. Data missing from registers but available from patient’s admission notes and antenatal (ANC) registers were also collected from these sources. All women who met the inclusion criteria were eligible to participate. For each birth record, socio-demographic information, maternal factors such as previous obstetric history (maternal age), neonatal information (sex, birth-weight) and delivery factors which included attendance by a skilled health worker, mode of delivery, and post-natal factors were extracted.

The main outcomes of this study were stillbirths and early neonatal deaths. Stillbirth was defined in accordance with World Health Organization definition of stillbirth for international comparison as death of a fetus weighing at least 500 g or after 22 completed weeks of gestation occurring before the complete expulsion or extraction from its mother [ICD-10]. Early neonatal death was defined as death that occurred within the first seven days of life. Data on late neonatal deaths could not be extracted as they are referred to local clinics for postnatal and therefore was not analysed.

Data from registers were double entered into Excel, cleaned and transferred to Stata 12.0 (Stata Corp, Texas, and USA) for analysis. Descriptive summaries were undertaken presenting frequencies, proportions, standard errors and associated 95% confidence interval for categorical characteristics while means, median, standard errors, *p*-values and associated 95% confidence interval for continuous characteristics.

### Ethical approval

Ethical clearance was granted by the University of Pretoria, Faculty of Health Sciences Research Ethics Committee as well as the Medical and Research Council of Zimbabwe. The need for consent was waivered by the Medical Research council of Zimbabwe. Confidentiality of records was adhered to and there were no personal identifiers used in the final report.

## Results

We sampled 427 records, 81 records were discarded due to more than 20% missing data. Of the discarded records, 3.2% had an adverse pregnancy outcome, 8.6% had complications and 6.2% had delivered by non-vertex delivery methods.

The remaining 346 records were used in the final analyses. Adverse pregnancy outcomes (stillbirth or neonatal death) were recorded in 54 (15.61%; 95% CI: 0.12–0.20) cases. Live births were recorded in 292 (84.39%; 95% CI: 0.80–0.88) cases.

Women sampled were between 17 and 43 years of age with a mean age of 26 (SD: 6.42). Most women (72.43%; 95% CI: 0.67–0.77) were between 20 and 34 years of age and 13.78% (95% CI: 0.10–0.18) were 35 years and older. Most of the women resided in urban areas (62.10%; 95% CI: 0.57–0.67) while 37.90% (95% CI: 0.33–0.43) were from rural areas and referred for maternal services (Table 1). Most of the women were married (97.92%; 95% CI: 0.95–0.99) while 2.08% (95% CI: 0.01–0.05) were separated, divorced or single. Close to 98% (95% CI: 0.964–0.999) of the women were Christians (either Pentecostal or orthodox, apostolic sect), and 2% (95% CI: 0.0004–0.0364) were non-Christians (either Moslem or African Tradition religion). Twenty two percent of the women (*n* = 73) had some form of primary education and more than 60 % had secondary education. Of the 346 women sampled, 12.7% (95% CI: 0.09–0.18) were formally employed as teachers, cashiers or nurse aides while 87.3% (95% CI: 0.82–0.91) were involved in informal trading or were housewives. Paternal variables were not analysed due to lack of data.

**Table 1:** Socio-demographic characteristics of women delivering at Sakubva hospital, January to June 2014. *p* < 0.05

Variable	N (%)	%	95% Confidence interval	p-value
Residential area	*n* = 343			0.761
Urban	213	62.10	0.57–0.67	
Rural	130	37.90	0.33–0.43	
Marital status	*n* = 288			0.596
Not married	6	2.08	0.004–0.037	
Married	282	97.92	0.96–1.00	
Maternal religion	*n* = 217			0.359
Non-Christian	4	1.84	0.0004–0.0364	
Christian	213	98.16	0.9635–0.9996	

### Maternal reproductive health characteristics

Sixty seven percent of women had no history of previous obstetric complications while 33% (95% CI: 0.27–0.38) had experienced a complication like stillbirth, neonatal death, abortion or eclampsia in a previous pregnancy (Table 2). Delivery by caesarean section in the previous pregnancy was also recorded as a previous complication. Diagnosis of a complication in previous birth resulted in precautionary measures like referral to next level for delivery.

**Table 2:** Reproductive, maternal, neonatal, and health system characteristics of women delivering at SDH, January–June 2014. p < 0.05

Variable	N (%)	%	95% Confidence interval	p-value
Maternal age	*n* = 341			0.132
< 20	47	13.78	0.10–0.17	
20–34	247	72.43	0.68–0.77	
35 _+_	47	13.78	0.10–0.17	
Obstetric history	*n* = 282			0.414
None	190	67.38	0.62–0.73	
Present	92	32.62	0.27–0.38	
HIV status	*n* = 303			0.825
Negative	257	84.82	0.81 -- 0.89	
Positive	46	15.18	0.11–0.19	
Neonatal sex	*n* = 333			0.361
Male	166	49.85	0.44–0.55	
Female	167	50.15	0.45–0.56	
Birth weight	*n* = 322			0.001
< 2500 g	47	14.60	0.11–0.18	
2500 g–4000 g	269	83.54	0.79–0.87	
> 4000	6	1.86	0.01–0.04	
Gestational age	*n* = 316			0.000
≥ 32	303	95.89	0.94–0.98	
< 32	13	4.11	0.02–0.06	
Parity	*n* = 334			0.630
0	109	32.63	0.28–0.38	
≥ 1	225	67.37	0.62–0.72	
Gravidity	n = 333			0.146
< 4	260	78.08	0.74–0.83	
≥ 4	73	2.92	0.17–0.26	
Birth attendant	*n* = 337			0.749
Unskilled	18	5.34	0.03–0.08	
Skilled	319	94.66	0.92–0.97	
Delivery complications	n = 334			0.000
None	121	36.23	0.31–0.41	
Present	213	63.77	0.59–0.69	
Delivery method	*n* = 338			0.000
NVD	260	76.92	0.72–0.81	
Non - NVD	78	23.08	0.19–0.28	
ANC visits	n = 80			0.075
Nulliparous	10	12.50	0.68–0.22	
1–3	29	36.25	0.26–0.47	
4+	41	51.25	0.46–0.62	

Fifteen percent of the women were HIV positive, while 2% (95% CI: 0.01–0.05) of the women had non-HIV chronic conditions like hypertension, diabetes, asthma, and psychosis prior to pregnancy.

Approximately 8% (95% CI: 0.03–0.18) of the women had an episode of malaria (*n* = 64) and 3% (95% CI: 0.004–0.1978) had tested positive for syphilis (*n* = 34) while pregnant. The median parity and gravidity were 1 and 2 respectively. The median inter-pregnancy interval (between the delivery under review and the previous birth) was 4 years (IQR 2–7).

### Pre-delivery

Of the 80 women who had information on ANC visits, 12.5% (95% CI 0.07–0.22) had not accessed antenatal care, while 36.25% (95% CI; 0.26–0.47) had 1 to 3 visits and 51.25% (95% CI; 0.40–0.62) had 4 or more visits. ANC visits were not assessed if conducted according to WHO Focused ANC recommendations as the information was missing.

### Neonate demographics

Fifty one percent of neonates were female and 49% (95% CI 0.44–0.55) male (Table 2). The interquartile weight range for neonates was 2700 to 3400 g with a mean weight of 3000 ± 599.26 g. The mean gestation age was 37 weeks while the minimum and maximum were 24 and 43 respectively. Approximately 15% (95% CL; 0.11–0.19) of the neonates were of low birth weight (< 2500 g) while 1.86% (95% CI; 0.01–0.04) had weight of above 4000 g. Birthweight was associated with adverse outcome (*p* = 0.001), where 31.91% of adverse outcomes where among the LBW neonates.

### Delivery factors

Most deliveries 94% (95% CI: 0.92–0.97) were attended to by a skilled health professional, midwife or doctor and the rest 6% (95% CI: 0.03–0.08) by non-skilled professionals. Skilled professionals were either midwifes or doctors as indicated in the register. All the non-skilled deliveries occurred at Sakubva, a secondary level facility. Reasons for delivery by non-skilled workers (e.g. nurse aides) at health centres could not be established in this study. Further studies to establish reasons for non-skilled delivery at facility need to be researched.

Seventy-six percent (95% CI: 0.72–0.81) of the women had a normal vertex delivery, 4.71% (95% CI: 0.03–0.08) successfully delivered a breech presentation baby through assisted NVD, and 18.21% (95% CI:0.15–0.23) delivered by caesarean section (Table 2). Firstly, 38 % of the women who had caesarean section and parity data available (*n* = 60), 38.3% were nulliparous and 61.67% were multiparity. Secondly, all caesarean section (*n* = 58) where among those with gestation age 32 and above. Thirdly, of the women who had a caesarean section performed, 22.97% where among those with complications. Finally, the overall reasons for conducting a caesarean section included hypertension in pregnancy, fetal distress, previous caesarean section and complications of a breech presentation.

Sixty-three, 63% (95% CI: 0.58–0.69) of women who delivered, experienced pregnancy associated complications. Complications recorded included, cord prolapse, mal-presentation, antepartum haemorrhage (APH), eclampsia, prolonged labor and fetal distress.

## Discussion

Prevalence of adverse pregnancy outcomes (stillbirths -macerated and fresh) and early neonatal deaths) at Sakubva hospital in Mutare district between January and June 2014 was 15.61% (95% CI: 0.12–0.20).

The high prevalence (15.61%) of adverse pregnancy outcomes is comparable to findings of other studies conducted in the region [19]. High neonatal mortality has been recorded in sub-Saharan Africa, Asia and Latin America, where approximately 25% of stillbirths result from delivery complications [20]. The stillbirth prevalence recorded in Jimma Hospital, an Ethiopian specialised hospital was 8% [21]. In Tanzania [13], the prevalence of stillbirths and intra-uterine deaths was reported as 18%. South Africa recorded a 13% prevalence rate of adverse pregnancy outcomes [22]. Most stillbirths occur during intra-partum in low-middle-income countries like Zimbabwe and are preventable [23]. Good quality essential maternal, neonatal health care must be incorporated into the health delivery system to decrease stillbirths and early neonatal deaths.

Birth weight, gestational age are contributors to adverse pregnancy outcomes. Low birthweight, and less than 32 weeks gestational age preterm neonates are more susceptible to hypoglycaemia, hypocalcaemia and hypothermia, which need to be managed to avoid death. Successful interventions to care for LBW and preterm babies include special baby care units (SBCU), exclusive breastfeeding and skin-to-skin care “kangaroo mother care.” Kangaroo mother care has been shown to substantially reduce neonatal mortality among LBW babies. However, Sakubva hospital has been practising kangaroo mother care since 2012, but the risk of death among the neonates is there, therefore this intervention needs to be intensified [24].

Delivery method and delivery complications were contributors to high adverse outcomes. The percentage of women who had a caesarean section in our sample was 18.21%. It is recommended that facilities provide caesarean section services to every woman who needs it reduces adverse outcomes. WHO guidance had set the caesarean section rates at 10–15% [25]. The need for caesarean section deliveries may be intricately linked to complications during pregnancy and birth, of which 22.58% (*p* = 0.00) of caesarean section in our study were among women who had complications. Thirty six percent of those that had a caesarean section had an adverse outcome. Morbidity associated with vaginal delivery of a breech presentation is caused by complications ranging from cord prolapse, aspiration of amniotic fluid to complications associated with difficulties of delivering the after-birth [24]. The Zimbabwean guidelines recommend caesarean section in a breech presentation baby if a woman is para 0, had previous caesarean section or when baby is big. In our study it was noted that about 4.73% (95% CI; 0.03–0.08) breech presentation delivered using assisted NVD method.

Intra-partum complications can result in fatal outcomes if not managed appropriately. Complications range from prolonged labour and fetal distress, associated with maternal and fetal conditions such as pre-eclampsia, eclampsia, abnormal presentation and cord accidents [26]. Programs targeted to improve these conditions pre- and intra-partum should influence birth outcomes.

Quality service provision through focused antenatal care is important in minimizing adverse pregnancy outcomes [15], especially if they can predict high risk deliveries. First: Our study recorded a 12.5% (*n* = 80) non-ANC booking. Quality ANC is essential in identification and management of infections such as malaria and syphilis, conditions like pregnancy induced hypertension and diabetes which can result in adverse outcomes. A study by Guzha et al. [27], showed a high primipara booking of 94.1% and a reduced maternal mortality in the group. However, Dodzo and Mhloyi [18] showed that perceptions of poor services, and high costs at health facility result in community deliveries. Zimbabwe hospitals require facility specific efforts to ensure provision of quality services. Secondly, of those that access ANC 36.25% had less than 4 visits while 51.25% had 4 or more visits. Focused ANC is a known strategy to improve maternity outcomes. The hospital should come up with strategies to improve focused ANC services. Lastly, high syphilis infection and HIV rates in our study needs monitoring. Mutare districts should explore methods that improve booking rates and quality of care throughout the continuum of care to reduce high prevalence of stillbirths and early neonatal deaths. Quality focused ANC though not a factor in our study, if improved will indirectly impact adverse outcomes by improving early identification of complications, resulting in appropriate intervention (method of delivery) and post-partum care.

Limitations to our study included the retrospective nature of the study, use of facility level data thus limiting accessible information to available parameters. The study is limited to descriptive nature and no analysis of factors associated with adverse outcomes could be assessed. About 19% of the 427 records were discarded due to lack of data (< 20%) which indicates poor record keeping at the facility. Malaria is endemic in the province (Manicaland) but our study only recorded 64 cases and no meaningful analysis could be conducted. Our findings in Mutare cannot be generalised to whole country. Notwithstanding these limitations, the study is a first step in description of adverse pregnancy outcomes in this low resource setting.

## Conclusion/recommendation

High prevalence rates of adverse pregnancy outcomes require targeted approaches. Quality essential maternal, neonatal health care must be incorporated into the health delivery system to decrease stillbirths and early neonatal deaths. Measures to minimize adverse pregnancy outcomes in Mutare district could include; 1. Intensified kangaroo care for low birthweight and pre-term neonates (< 32 gestation) 2. the facility should ensure provision of quality prenatal care during focused antenatal care which will ensure identification of complications, infections like HIV and syphilis and manage or refer appropriately; 3. Offer appropriate delivery methods by the provision of caesarean section to women in need. Continuous assessment and management of breech delivery and complications is recommended. Finally, further studies into factors associated with adverse outcomes are recommended.

## Abbreviations

ANC: Antenatal Care
APH: Antepartum Haemorrhage
HIV: Human Immunodeficiency Virus
LBW: Low Birth Weight
MDG: Millennium Development Goal
MICS: Multiple Indicator Cluster Survey
NMR: Neonatal Mortality Rate
NVD: Normal Vertex Delivery
PIH: Pregnancy Induced Hypertension
RBF: Result Based Financing
ZDHS: Zimbabwe Demographic Health Survey
